# Impact of Active Disinvestment on Decision-Making for Surgery in Patients With Subacromial Pain Syndrome: A Qualitative Semi-structured Interview Study Among Hospital Sales Managers and Orthopedic Surgeons

**DOI:** 10.34172/ijhpm.2023.7710

**Published:** 2023-08-13

**Authors:** Timon H. Geurkink, Perla J. Marang-van de Mheen, Jochem Nagels, Rudolf W. Poolman, Rob G.H.H. Nelissen, Leti van Bodegom-Vos

**Affiliations:** ^1^Department of Orthopaedics, Leiden University Medical Center, Leiden, The Netherlands; ^2^Department of Biomedical Data Sciences, Medical Decision Making, Leiden University Medical Center, Leiden, The Netherlands

**Keywords:** Active Disinvestment, Subacromial Pain Syndrome, Low-Value Care, Medical Overuse

## Abstract

**Background:** Withdrawal of reimbursement for low-value care through a policy change, ie, active disinvestment, is considered a potentially effective de-implementation strategy. However, previous studies have shown conflicting results and the mechanism through which active disinvestment may be effective is unclear. This study explored how the active disinvestment initiative regarding subacromial decompression (SAD) surgery for subacromial pain syndrome (SAPS) in the Netherlands influenced clinical decision-making around surgery, including the perspectives of orthopedic surgeons and hospital sales managers.

**Methods:** We performed 20 semi-structured interviews from November 2020 to October 2021 with ten hospital sales managers and ten orthopedic surgeons from twelve hospitals across the Netherlands as relevant stakeholders in the active disinvestment process. The interviews were video-recorded and transcribed verbatim. Inductive thematic analysis was used to analyse interview transcripts independently by two authors and discrepancies were resolved through discussion.

**Results:** Two overarching themes were identified that negatively influenced the effect of the active disinvestment initiative for SAPS. The first theme was that the active disinvestment represented a "Too small piece of the pie" indicating little financial consequences for the hospital as it was merely used in negotiations with healthcare insurers to reduce costs, required a disproportionate amount of effort from hospital staff given the small saving-potential, and was not clearly defined nor enforced in the overall healthcare insurer agreements. The second theme was "They [healthcare insurer] got it wrong," as the evidence and guidelines had been incorrectly interpreted, the active disinvestment was at odds with clinician experiences and beliefs and was perceived as a reduction in their professional autonomy.

**Conclusion:** The two overarching themes and their underlying factors highlight the complexity for active disinvestment initiatives to be effective. Future de-implementation initiatives including active disinvestment should engage relevant stakeholders at an early stage to incorporate their different perspectives, gain support and increase the probability of success.

## Background

**Key Messages**

**Implications for policy makers**

- Based on the results of this study, the effectiveness of an active disinvestment initiative seems to be largely dependent on the support for active disinvestment by relevant stakeholders.
- To gain support for active disinvestment and improve the probability of success, policy-makers should actively engage relevant stakeholders early on in the development of the disinvestment strategy.
- In specific, active disinvestment initiatives must have sufficient saving-potential and a required effort from hospital staff that is proportionate to the financial impact, need to be clearly defined and enforced in overall hospital agreements, and be supported by evidence and guidelines.

**Implications for the public**

The withdrawal of reimbursement for low-value procedures through a policy change, ie, active disinvestment, is considered a potentially effective but underused strategy to reduce low-value care. This study found that the effectiveness of such initiatives seems to be largely dependent on the support for active disinvestment from relevant stakeholders (eg, hospital sales managers and orthopedic surgeons). Therefore, policy-makers should engage relevant stakeholders early on in the development of an active disinvestment initiative to improve the possibility for success. Furthermore, several specific factors were identified within this study that may contribute to the limited support for active disinvestment by relevant stakeholders.

Healthcare costs have increased drastically worldwide over the past several decades and are expected to continue rising in the coming years.^1^ This rise in healthcare costs forces policy-makers to explore solutions to ensure good quality of care while working with limited financial resources.^2,3^ One potential solution to solve this challenge is to reduce low-value care, ie, services for which there is little evidence of benefit for patients or that cause more harm than benefit (eg, risk of complications, psychological distress, treatment burden and financial loss).^4^ Currently, it is estimated that approximately one-third of all medical spending is related to low-value care.^5^

 Choosing Wisely (CW) is an international campaign launched to open the discussion on low-value care and develop interventions to reduce overuse.^6-8^ However, the literature merely shows a slight decline or unchanged trends in low-value care following such CW campaigns.^9,10^ Smaller reductions in the use of low-value care are associated with the release of CW recommendations than for a policy change eliminating reimbursement as shown recently for low-value use of vitamin D screening.^11^ Therefore, withdrawal of reimbursement through a policy change — or active disinvestment — has been suggested as a promising alternative.^12^ Active disinvestment has been associated with substantial reductions in low-value care and is considered an effective but underused de-implementation strategy.^11,13^ However, it is also considered a very complex strategy, influenced by various potentially complicating factors (eg, level of support for disinvestment among clinicians and policy-makers), which make successful disinvestment a complex undertaking.^12,14,15^

 Given that less than half of the disinvestment initiatives have been successful until now,^16^ more research is needed to further explore and understand the complex mechanism through which active disinvestment may have an effect on reducing low-value care.^16-18^ Theoretical frameworks that may facilitate understanding how active disinvestment influences (clinical) decision-making of different stakeholders for specific interventions are lacking but needed to guide future active disinvestment initiatives.^18,19^ Therefore, we investigated how the active disinvestment initiative of subacromial decompression (SAD) surgery for subacromial pain syndrome (SAPS) in the Netherlands influenced clinical decision-making around surgery, including perspectives of hospital sales managers and orthopedic surgeons, to increase our understanding on how active disinvestment initiatives may exercise their effect on clinical decision-making.

## Methods

###  Study Design 

 A qualitative study was conducted with semi-structured interviews among both hospital healthcare sales managers as well as orthopedic surgeons treating SAPS patients. We used a qualitative research approach as this provides more in-depth insights into processes that numerical data cannot capture and is able to fully explore the perspectives of relevant stakeholders.^20^ All results were reported according to the COnsolidated criteria for REporting Qualitative research (COREQ) checklist.^21^

###  Setting

 The Netherlands has a private-public financed healthcare system, with mandatory standard private healthcare insurance for all Dutch citizens from healthcare insurance companies and optional additional insurance (eg, special dental care).^22^ The insurance market is dominated by four large insurers who together have a total market share of approximately 85%.^23^ Most insurers operate nationally, but market shares vary per region and each region has a different market leader.^23^ The government has given healthcare insurers an essential role in quality assurance by allowing them to selectively contract healthcare providers (eg, hospitals) and specific interventions.^24^ This selective contracting by healthcare insurers aims to reduce overall healthcare costs and improve the hospitals’ quality of care. Periodically (mostly each year), healthcare insurers negotiate with hospitals (through their hospital sales managers) on prices and volumes of specific interventions. The latter gives the healthcare insurer the opportunity to apply active disinvestment initiatives for low-value care interventions and thereby reduce their costs.

###  Description of Intervention

 SAD surgery for SAPS is considered a low-value care intervention as high-quality literature found no overall clinical benefit of surgical treatment for SAPS compared to non-operative treatment.^25-28^ Nevertheless, in 2016 still approximately 10 000 patients underwent SAD surgery for SAPS in the Netherlands.^29^ Therefore, one of the four largest Dutch healthcare insurers introduced an active disinvestment initiative from January 2020 onwards to reduce SAD surgery in SAPS patients. This healthcare insurer considered 80% of all currently performed surgical procedures for SAPS to be low-value care. To reduce the use of this low-value procedure, this insurer decided to contract 30% fewer surgical procedures for SAPS from each contracted hospital compared with the number of procedures in the previous year. The insurer informed hospitals about this specific active disinvestment by email and during the annual healthcare contract negotiations with hospital sales managers.

###  Participant Selection 

 As the active disinvestment initiative of the healthcare insurer primarily targeted hospitals, we approached a purposive sample of 25 different relevant stakeholders working withing these hospitals (ie, hospital sales managers and orthopedic surgeons) to participate in the semi-structured interviews. In the Netherlands, hospital sales managers form the direct link between hospitals and healthcare insurers. They are responsible for making financial arrangements on reimbursement of healthcare services provided to patients by a hospital. In addition, they are accountable for communicating healthcare insurers’ policy changes within the hospital, including active disinvestment initiatives. Therefore, they are considered key players in making the process of active disinvestment work in daily practice. The orthopedic (shoulder) surgeons treating SAPS patients were interviewed as they are ultimately responsible for clinical decision-making together with the patient.

 We purposively sampled participants from different types of hospitals, ie, academic and non-academic teaching and non-teaching hospitals or independent treatment centers (ITCs), and different geographical regions because the impact of active disinvestment may vary significantly between types of hospitals and regions, depending on which part of their patients is insured by the healthcare insurer applying the active disinvestment initiative. The relevant stakeholders were recruited from the authors’ professional network. All stakeholders were invited to participate and received information about the interview by email. Twenty of the 25 contacted stakeholders (80%) agreed to participate. One orthopedic surgeon did not agree to be interviewed due to a lack of time, while four approached stakeholders (ie, one hospital sales manager and three orthopedic surgeons) did not respond to the invitation, despite several reminders by email.

###  Data Collection

 Given their different role in the decision-making process, separate interview guides were created for hospital sales managers and orthopedic surgeons (Supplementary file 1). All interviews started with the question whether the participant was familiar with the active disinvestment for SAD surgery among SAPS patients from the particular healthcare insurer. The interviewer (THG) explained the active disinvestment initiative if they were unfamiliar with this. From this point, the interviews for hospital sales managers included the following topics: (*i*) the negotiation process between healthcare insurers and healthcare providers, (*ii*) the attention given to the active disinvestment initiative for SAPS during these negotiations, (*iii*) the consequences of this active disinvestment for the hospital, and (*iv*) the perceived effect of this active disinvestment on clinical decision-making. The interviews with orthopedic surgeons covered the following topics: (*i*) their treatment strategy for SAPS, (*ii*) the surgeons’ perspectives about the active disinvestment initiative, and (*iii*) the perceived effect of the active disinvestment on clinical decision-making. Potentially relevant factors (related to organizational context or individual professional) that might influence how the active disinvestment worked were taken from a study by van Dulmen et al evaluating barriers and facilitators to reduce low-value care, and added to the interview guide as topics to discuss during the interview.^30^ Participants were actively stimulated to say everything that came to mind and share their experiences and opinion. At the end of the interview, all participants had the opportunity to provide additional feedback.

 Since the COVID-19 pandemic hindered face-to-face contact all semi-structured interviews were conducted and video-recorded (after verbal consent was obtained) via secured video calls (Microsoft Teams) by the same interviewer between November 2020 and October 2021. The interviewer (THG, male), a physician with additional qualitative interviewing training, did not have an established relationship with the participants before the interview nor was involved in clinical care. Two pilot interviews were conducted with one orthopedic surgeon and one hospital sales manager to test relevance and refine the interview questions. Because the pilot interviews did not result in significant changes in the interview guide, both interviews were included in the analysis. The interviews continued until data saturation was reached, defined as at least three consecutive interviews revealing no new insights.^31^ The median interview duration was 34 minutes (interquartile range: 30-39 minutes). During the interviews, the interviewer took notes to direct further questioning. Repeat interviews were not conducted and transcripts were not returned to the participants for comment or correction. Participants did not receive any financial compensation for their time.

###  Data Analysis

 All interviews were transcribed verbatim and entered into ATLAS.ti (version 7.0). THG and LvBV verified transcript accuracy. Interview transcripts were analysed using inductive thematic analysis. Inductive thematic analysis was applied to increase our understanding how the active disinvestment strategy may exercise its effect on clinical decision making in daily practice, thereby contributing to further development of theory rather than testing an existing theory. Thematic analysis is a flexible approach that identifies patterns within qualitative data, which is especially useful for describing processes that lack an existing theoretical framework.^32^ After familiarizing with the data, initial codes were identified and a coding tree was developed into which the data was assigned. All interviews were independently coded by two authors (THG and LvBV). Discrepancies were discussed until a consensus was reached. Coded text segments were searched for and grouped into overarching themes by THG and LvBV. Overarching themes were defined as a group of factors that might influence how active disinvestment would affect clinical decision-making on SAD surgery for SAPS. The analysis of overarching themes was iterative and continuous throughout data collection. The overarching themes were inspected and discussed by THG, PJMvdM, and LvBV for recurring themes and influencing factors on the disinvestment process until consensus was reached. Participants did not receive the results of the analyses and were not asked to provide feedback on the findings.

## Results

 Ten hospital sales managers and 10 orthopedic surgeons from 12 different hospitals were interviewed, with data saturation achieved after respectively nine and eight interviews. The healthcare insurer applying the active disinvestment initiative had the largest market share in three (25%) hospitals. Most (55%) interviewed participants worked in non-academic teaching hospitals. Three hospital sales managers (30%) and four orthopedic surgeons (40%) were familiar with the active disinvestment initiative for SAPS patients prior to the interview. Descriptive characteristics of the respondents are shown in Table 1.

**Table 1:** Baseline Characteristics of Study Participants

**Participant Characteristics**	**Hospital Sales Managers (n = 10)**	**Orthopedic Surgeons (n = 10)**
Mean Age (SD)	46 (8.1)	50 (7.8)
% Female	50	20
Mean years of experience as orthopedic surgeon (SD)	NA	13 (7.2)
Mean number of SAPS patients per week (SD)	NA	13 (9.1)
Hospitals		
Academic	1	1
Non-academic teaching	5	6
Non-academic non-teaching	3	3
Independent treatment center	1	0
Healthcare insurer market leader in hospital	3	3
Familiar with active disinvestment for SAPS patients	3	4

Abbreviations: SAPS, subacromial pain syndrome; SD, standard deviation; NA, not available.

 Thematic analysis resulted in the identification of two overarching themes which negatively influenced the support for the active disinvestment of SAD surgery for SAPS patients. The first theme was that the active disinvestment represented a ‘Too small piece of the pie’ for the hospitals. Particularly hospital sales managers stated that the active disinvestment initiative for SAPS (*i*) had little financial consequences for the total hospital budget, (*ii*) was only part of the negotiation process in the sense of that healthcare insurance companies used it merely to lower the overall pricing of the hospital’s overall contract agreement, (*iii*) required too much effort from hospital staff to accomplish only a slight reduction in overall costs, and (*iv*) was not clearly defined. For these reasons the active disinvestment did not influence hospital-level decision making and information regarding the active disinvestment was not communicated within the hospital to orthopedic surgeons (see Figure).

**Figure:**
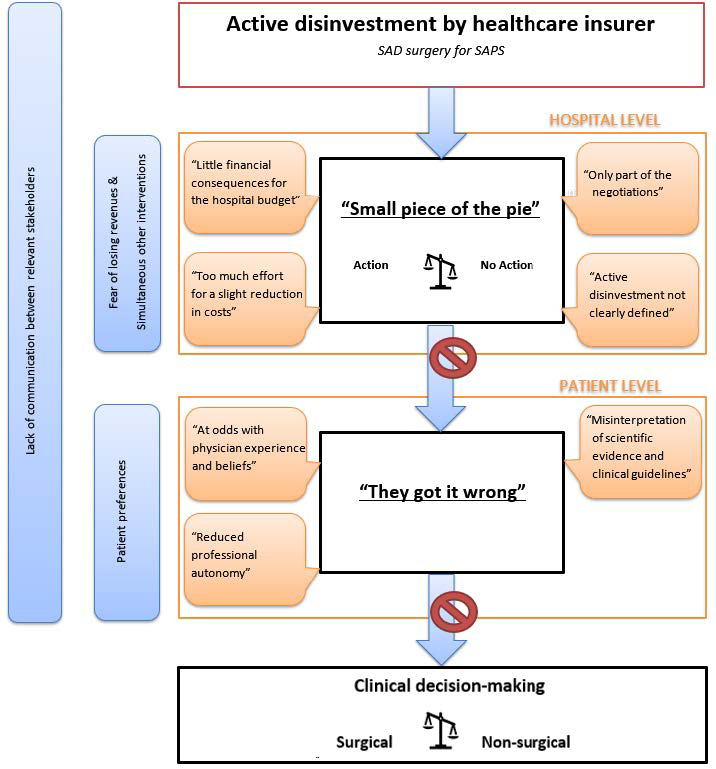
Factors negatively Influencing the Effectiveness of the Active Disinvestment Initiative on Clinical Decision-Making. Abbreviations: SAD, subacromial decompression; SAPS, subacromial pain syndrome.

 The second overarching theme was ‘They [the healthcare insurer] got it wrong.’ This theme was mainly highlighted by orthopedic surgeons who disagreed with the active disinvestment by the healthcare insurer. More specifically, the surgeons reported that the active disinvestment initiative was (*i*) the result of misinterpretation of scientific evidence and clinical guidelines, (*ii*) at odds with physician experience and beliefs, and (*iii*) reduced the professional autonomy of clinicians. With regard to physician experience and beliefs, the general obligation as a physician to provide care was highlighted, as well as that SAD surgery could still be beneficial for specific patients and should therefore remain as a treatment option. For these reasons the active disinvestment did not influence patient-level decision making (see Figure).

 Besides these two overarching themes, several other contextual factors were identified that also influenced the effectiveness of the active disinvestment, such as a lack of communication between relevant stakeholders, patient preferences, fear of losing revenues and simultaneous other interventions that rewarded rather than penalized hospitals for not performing specific procedures. The overarching themes, subthemes and contextual factors are described in the following section, with representative quotes supporting each theme shown in Table 2. Although most participants indicated they were not against active disinvestment initiatives, we did not identify any facilitating factors for the active disinvestment initiative to work as intended to reduce SAD surgery for SAPS.

**Table 2:** Representative Quotations Supporting Each Theme

**Subtheme**	**Quote Number**	**Representative Quotations**
**“Too Small Piece of the Pie”**		
*Little financial consequences for the hospital budget*	1	“I mean: there is so much money involved. So let’s then focus on the big groups, on the mass and on the good things. This incentive to me comes across as a specialization within a specialization within a sub-specialization” (SM 3).
	2	“Look, with the healthcare insurer we negotiate about 100 million euros. Yes, you know, then you are not going to talk about one specific diagnosis treatment combination, that does not happen” (SM 8).
	3	“I am not going into details, because that would go beyond what I am allowed to do. I cannot describe the details of such an agreement here. But it had no real financial impact on the total of the agreement” (SM 4).
	4	“According to me is that not our large group [of patients] but I don’t know that myself. In other words, then you will not notice that at all, if you only have a fraction of [patients insured by] [Name insurer]” (OS 2).
*Only part of the negotiations*	5	“Sometimes it seems as if you have a lot of conversations, where there is only push to knock off a little of the overall price” (SM 4).
	6	“Because you have to realize that during the negotiation this is only one part of the total amount on the table. In the end, after exchanging a lot of arguments, you come to an agreement with each other, which is often painful on all fronts. You have to leave certain things to be able to come to an agreement. This is also to the case for the healthcare insurer. They too cannot achieve everything from their dreamed mandate” (SM 4).
	7	“It is often under one big insurance ceiling, so then you get sort of a waterbed effect. So if you say: we want you to do this much fewer procedures and if you don’t entirely exclude that from the contract, so take it out from the ceiling in a separate financial agreement, then it may be possible that you can fill it up with for instance acute care or birth care. So the only way a healthcare insurer can say that you cannot do it anymore and we are going to enforce it, is that they cut out the entire block and really make it a separate part of the agreement. With a clear ceiling on the procedure so that it is really impossible to reimburse it anymore” (SM 6).
	8	“There is often discussion about this during negotiations, with a lot in plus and a lot in minus, then these die individually so to speak and you come to an overall deal, 30, 40, 50, 60 million, in which everything is quasi intertwined” (SM 7).
	9	“And with that (with the active disinvestment) you are actually forced to stop performing that procedure as you don’t get paid for it. That works I think only limited because what you do is that you often agree on one big pocket of money in which you apply the reduction. But if you secretly keep performing that procedure and instead do not perform something else where you have not talked about, then you will still receive the money but for the wrong things” (SM 1).
*Too much effort for a slight reduction in costs*	10	“You are talking about a legitimate 420 people, that is a very small number [of patients] and if you also take off the ones with the two other indications then that leaves so few, that yes, is it really worth the effort to spend time on it?” (OS 9).
	11	“And let’s then mainly talk about the good things and not about a few niches which will take 80 percent of the energy, but only account for 2% of the costs” (SM 3).
	12	“At the moment that you would you want to take steps towards efficiency, then you are looking for the big hits. Because you have to look at it this way, the commitment of people and resources is probably just as much to improve a certain procedure where we do only a small amount, compared- to where we do large amounts …. But more from the perspective: how do I invest my resources that are limited, human capacity too, to monitor [this]” (SM 1).
	13	“All the topics that are now on the orthopedics agenda. Yes, these have passed here in the last few years. Still I am forced to catch up on a certain indexation and to make a plan. For which I actually need to hire someone, who would write than plan for me. Whilst we are already doing this…. you are almost forced to go along in that flow. While in the end it often doesn’t lead to the intended goal, or that goal has already been achieved. It involves a lot of effort” (SM 10).
*Active disinvestment not clearly defined*	14	“But what we find very difficult about that information from [Name healthcare insurer] to which you just referred. That is that they indeed say: yes, 80 percent [of these procedures] you should no longer- do. But you don’t really know what 100 percent is. That I find very difficult as you only see what you do, maybe that is already very little, that you have left already that 20 percent [of the procedures that is of value to the patients].... So yes, what has actually been your baseline measurement?” (SM 5).
	15	“We expect that next year you only do one-third of this [number of procedures]. What kind of discussion is that?” (SM 9).
	16	“If a healthcare insurer would really shut it down with us and would say: you just cannot reimburse this [procedure] anymore, because it is not meaningful and that has been proven in so much literature. You should not do this anymore. Then we would send out a very clear signal [to the clinicians] you are not allowed to do this anymore” (SM 6).
**Subtheme**	**Quote Number**	**Representative Quotations**
**“They Got it Wrong”**		
*Misinterpretation of scientific evidence and clinical guidelines*	17	“Yes, I’m fine with that [active disinvestment strategy by insurer] as long as they are well-founded and that’s where it goes wrong. They don’t have the knowledge, of course they have some medical advisors, but they don’t have substantive knowledge to enter a discussion with me. They don’t have the clinical experience with these patients and can’t interpret scientific research” (OS 1).
	18	“Regardless of the situation, I don’t think the overall trend is good. Maybe it’s typical doctors reasoning but they [healthcare insurers] can’t gauge the true value of the science. They are good with numbers and at negotiating but not in valuing scientific research and its relation with clinical practice” (OS 8).
	19	“And certainly the SAPS complaint, that is such a diverse group of patients so that you can hardly draw any conclusions about SAPS treatment in general” (OS 2).
	20	“They [healthcare insurer] include all kinds of things under the SAPS diagnosis…. So medically speaking, those are all very different things. But the healthcare insurer, the layman, includes everything under the same as if it is one big umbrella diagnosis, covering everything” (OS 4).
	21	“I know that they have been working on that for a long time, to stop reimbursing this care, however, they classify everything under one diagnosis. I think that you can’t do that, an acromioclavicular resection is suddenly a part of SAPS. That’s not in our guideline, that’s just not right” (OS 5).
	22	“In line with this, my biggest fear is that they will also stop reimbursement for cuff repair as they will say it is the same. But it’s not the same. They classify everything under the SAPS syndrome, one collective term for every diagnosis. Yes, before you know it you can’t perform any surgery anymore. Not that that’s what this is about, but I think it’s a worrying development” (OS 8).
	23	“Guidelines remain guidelines and it is not the case that this means that these interventions must absolutely not be performed and it is also not said that it’s a medical error when you perform this procedure. Therefore, I don’t understand why this policy is used by the healthcare insurer” (OS 7).
*At odds with physician experience and beliefs*	24	“When they say: it is not allowed anymore, it will no longer be reimbursed. Yes, then I’ll use a different [reimbursement] code as I still have a patient who is crying out in pain. I have a general duty of care to help the patient. It is very strange that they say that I can’t do this. They should not be able to do this. They also have a general obligation to provide care” (OS 9).
	25	“When I notice that my treatments don’t have any effect, than I’m not going to offer it. The majority of patients that we treat are eventually happy and have less pain. I don’t care whether this is the result of surgery, an injection or some explanation. If I can treat patients well with conservative treatment then I will be happy to do so. But I still think there is a role for surgical treatments” (OS 2).
	26	“But when there is a persistent problem and the patient has had adequate treatment for at least one year, our physical therapists cannot do anything anymore, the diagnosis is confirmed on echo and a subacromial injection provides temporary pain relief, and everything points towards that direction [SAPS]. Yes, then I think you should still have the option to perform surgery” (OS 5).
	27	“When I think there is a medical indication, then that’s it and then I’m going to do that. I can still justify this for myself from the medical evidence. But on the other hand, it’s so artificial to say: we stop reimbursement. Look, orthopedic surgeons are smart enough to use another code [diagnosis and procedure code combination]. So yeah, this is not a good way to regulate something at all” (OS 7).
*Reduced professional autonomy*	28	“Well, I think that the healthcare insurer should not take the place of the doctor…. I know for sure that we, orthopedic surgeons, are not waiting for healthcare insurers that tell us what we should and shouldn’t do” (OS 7).
	29	“Speaking for myself, I have the feeling that they think that we want to fool someone or to get financial gain out of something. The more subacromial decompressions we do, a simple procedure, the more money we earn. But nobody, at least none of my colleagues, thinks about this while doing consultations at the outpatient clinic” (OS 6).
	30	“I think that the incentive should be initiated by the professional association…. In the end, I think that clinicians should decide and that the treatment policy should not be determined by the health insurer” (OS 8).
	31	“You get people in the right direction more quickly if they are intrinsically motivated, instead of extrinsic matters. I think that the social control in the Netherlands is also large enough. At a certain point, you know from colleagues if they still do it [procedures] or if they do it a lot and you come across them and you will see their patients for a second opinion. So, I think the circuit also works well and you don’t want to be seen as the one who is still performing these operations. I think everyone has his own pride in that. I think that this might work better than a healthcare insurer interfering in this, as they are not seen as a partner. And then I’m putting it mildly” (OS 1).
**Subtheme**	**Quote Number**	**Representative Quotations**
**Contextual Factors**		
*Lack of communication between relevant stakeholders*	32	“What I sometimes find rather peculiar about [name healthcare insurer], they are, in my opinion, very good at just throwing things over the fence…. If you really want to change something, then start a conversation” (SM 3).
	33	“We are never told about this by sales or the medical manager, that we need to change certain things in our working procedure” (OS 2).
	34	“Within our group [orthopedic surgeons] we have discussed this extensively and I also formulated a response [for the healthcare insurer], which was checked by the others.… The stupid thing is, I don’t hear anything about it. I know that this now also is a point of concern nationally, so that it will also be tackled nationally after that discussion in the shoulder elbow working group. But I haven’t heard back what’s going to happen now, whether the care we provide will be reimbursed or not” (OS 5).
*Patient preferences*	35	“People who have had the [same] surgery in the past, had good results on that side and now have similar pain or shoulder complaints on the other side. They want surgery” (OS 3).
	36	“By the way, then people will sometimes just go to Belgium. And that will subsequently also be reimbursed by the insurers. So there are patients who bypass the system” (SM 4).
*Fear of losing revenues*	37	“But the projects [reducing low-value procedures] don’t run themselves as the surgeons are financially rewarded for their volume [of performed procedures]. Therefore, at the moment you remove volumes, they will not be the first ones applauding for you” (SM 2).
	38	“We are a hospital in which incomes and honoraria are highly correlated with production. So yes, I dare to state that these kind of desired movements will not be helped by the way in which hospitals like ours work and that such incentives could be very tricky in that context.” (SM 5).
	39	“I understand that there are still clinics where they [surgeons] just do everything: hips, knees, shoulders, and that subacromial pain leads to a subacromial decompression…. Because they make their living with this. An arthroscopy will give you a lot of money. So if you just do a bursectomy or a Neer acromioplasty, that will yield a lot of money” (OS 9).
*Simultaneous other interventions*	40	“For example, there is now also the ZE&GG program^a^, we actively started with this last year…. Last year this was a bit more noncommittal and you could see this in the varying degree of involvement between departments. This year we are going to make it mandatory as there are financial agreements linked to this for the hospitals” (SM 3).
	41	“I’m not familiar with the example [active disinvestment strategy for SAPS], that’s how I should phrase it. However, the general tendency and the conversations, we are constantly working on this. Patients a day shorter. They used to spend three days in a room after surgery, now only two or sometimes one. So in general, we are already working on similar trajectories” (SM 8).
	42	“Then we’re talking again about the ZE&GG agenda. The hospitals get a certain additional compensation for increases in wages and collective labor agreement, and for that they have agreed to actively participate with that ZE&GG agenda…. So in that sense this works with an incentive. Apparently, the hospitals were interested and have agreed with this. Because they get their money, but also do something in return. We all get it. I think this might be relevant for you to consider, to what extent would incentives work better than disincentives” (SM 1).

Abbreviations: SM, hospital sales manager; OS, orthopedic surgeon; SAPS, subacromial pain syndrome. ^a^ The ZE&GG program is a program for the evaluation and appropriate use of care from Zorginstituut Nederland. Within this program, hospitals work together with healthcare insurers and the Dutch government in order to actively reduce the use of low-value care. The hospitals receive a financial incentive for their participation.

###  Too Small Piece of the Pie

####  Little Financial Consequences for the Hospital Budget

 Both hospital sales managers and orthopedic surgeons stated that SAD surgery for SAPS reflects an insignificant part of the total care provided by the hospital. As a result, the active disinvestment for one specific procedure represented little financial value compared to the overall costs of care provided by the hospital (Quote 1-2). The active disinvestment initiative in its current form was considered to have no financial consequences for the hospitals’ budget, especially in hospitals where this healthcare insurer only had a small market share (Quote 3-4).

####  Only Part of the Negotiations

 Hospital sales managers mentioned that the active disinvestment was only a tiny part of the negotiation process between the healthcare insurer and hospitals. They believed it merely aimed to lower the price of the overall contract agreement rather than explicitly reducing the number of performed surgeries for SAPS patients (Quote 5-7). At the end of the negotiation process, a contract is drawn up that includes the total volume of procedures (not only SAD for SAPS, but one overall agreement containing all procedures within the hospital) together with an overall price (Quote 8). Within this agreement, the active disinvestment no longer receives any particular attention, thereby leaving the possibility to perform SAD surgery for SAPS and receive reimbursement for it (Quote 9).

####  Too Much Effort for a Slight Reduction in Costs

 Related to the first sub-theme that SAD surgery for SAPS had little financial consequences for the hospital, both hospital sales managers and orthopedic surgeons believed the amount of effort they had to put into reducing SAD surgery was disproportionate to the saving-potential (Quote 10-11). They also indicated that efforts by hospital staff to reduce low-value care procedures are likely to be the same for low-volume procedures as high-volume procedures. From an efficiency perspective, hospital staff should therefore better focus on high-volume procedures (Quote 12). In addition, hospitals often already have initiatives that aim to reduce the use of low-value care procedures so that such initiatives from healthcare insurers lead to unnecessary duplication of work (Quote 13).

####  Active Disinvestment Not Clearly Defined

 A final sub-theme was that the active disinvestment was unclear (eg, the use of relative outcome measures without adequate baseline measurement), not specific enough and still allowed to perform surgery for SAPS as part of the surgeries were still reimbursed (Quote 14-15). Hospital sales managers argued that surgeons would only stop performing SAD surgery for SAPS when there would be no reimbursement at all (Quote 16).

###  They Got it Wrong

####  Misinterpretation of Scientific Evidence and Clinical Guidelines

 Orthopedic surgeons felt the healthcare insurer had misinterpreted the existing scientific evidence and clinical guidelines on which the active disinvestment was based. In general, they supported the reduction of low-value care. Still, they highlighted that healthcare insurers often lack the knowledge, skills and clinical experience to correctly interpret the scientific evidence and guidelines, so that active disinvestment initiatives cannot be based on their interpretation (Quote 17-18). Given that SAPS is an umbrella diagnosis covering a heterogeneous group of etiologies with different treatment needs, they felt that too many diagnoses and procedure codes were included in this particular active disinvestment. As consequence, the active disinvestment did not correctly reflect the SAPS population for which surgery is or is not appropriate, nor which surgical procedures were not appropriate for these patients (Quote 19-22). In addition, clinical guidelines aim only to guide clinical decision-making, and do not dictate treatment for specific patient groups. After all, it is the health professional’s decision to decide on an individual patient’s treatment given the specific input of clinical information of an individual patient in conjunction with the clinical experience of the orthopedic surgeon. Thus, surgeons felt that clinical guidelines should not be used to formulate active disinvestment initiatives (Quote 23).

####  At Odds With Physician Experience and Beliefs

 Orthopedic surgeons argued that withholding treatment options resulting from active disinvestment initiatives, is at odds with their general obligation to provide care (Quote 24). They also declared that healthcare insurers have a similar obligation, to reimburse care needed by the patient, which is also violated by this active disinvestment. In addition, surgeons disagreed with the active disinvestment initiative as they believed that some patients could still benefit from surgery, often based on previous individual experience (Quote 25-26). They were not convinced that such a disinvestment initiative would result in a reduction of surgery for SAPS as surgeons would still decide to perform surgery when deemed appropriate by changing their coding practices rather than not doing the surgery anymore (Quote 27).

####  Reduced Professional Autonomy

 Orthopedic surgeons argued they had extensive training to weigh different treatment options appropriately, to best care for their patients. Applying such active disinvestment initiatives, the healthcare insurer intervenes in the physician’s work by limiting clinical treatment options and thereby diminishes their professional autonomy. The surgeons mentioned that healthcare insurers should not take over the role of physicians (Quote 28) and felt that such initiatives expose an underlying mistrust of healthcare insurers in the professional autonomy of physicians (Quote 29). In general, they stated that healthcare insurers should not initiate such initiatives, but that these should be initiated by the orthopedic professional association (Quote 30-31).

###  Contextual Factors

 Four contextual factors were identified that influenced the effectiveness of the active disinvestment initiative for SAPS. First, both hospital sales managers and orthopedic surgeons mentioned the lack of communication between relevant stakeholders. More communication was needed between the healthcare insurer and hospitals as sales managers needed additional explanation about the active disinvestment by the healthcare insurer (Quote 32). Increased communication was also required among the relevant stakeholders within hospitals as orthopedic surgeons indicated not being informed about the active disinvestment by neither the sales managers nor the healthcare insurer (Quote 33-34). Second, patient preferences may persuade orthopedic surgeons to perform surgery for SAPS (Quote 35-36). Third, fear of losing revenues was suggested as a contextual factor as orthopedic surgeons may have financial benefit from performing surgery, which might reduce the impact of the active disinvestment (Quote 37-39). Striking was that these were only suggested to apply to others (eg, sales managers about orthopedic surgeons, or orthopedic surgeons working in general hospitals about surgeons working in ITCs) and therefore it remains unclear whether this really affects active disinvestment. Finally, simultaneous other interventions (either national or within hospitals) aiming to reduce low-value care may also influence how well the active disinvestment will work (Quote 40-41). Some of these interventions financially rewarded hospitals for not performing low-value care procedures anymore. Sales managers thought that such initiatives would be more successful than active disinvestment as this would be more motivating for hospitals (Quote 42).

## Discussion

 The present study showed that two overarching themes negatively influenced the impact of the active disinvestment regarding SAD surgery for SAPS in the Netherlands, as both hospital sales managers and orthopedic surgeons did not support the active disinvestment from the healthcare insurer. Particularly hospital sales managers felt it represented a “Too small piece of the pie” where it was merely used in negotiations to reduce costs but had little financial consequences for the hospital budget while requiring a lot of effort, and was not clearly defined nor enforced in the overall agreements between healthcare insurers and hospitals. As a result, they did not communicate the information on the active disinvestment initiative to orthopedic surgeons. Additionally, orthopedic surgeons felt “They got it wrong” as the active disinvestment had incorrectly interpreted the evidence and guidelines, was at odds with physicians’ experiences and beliefs, and perceived it as a reduction in professional autonomy. As a result, it did not affect their clinical decision-making regarding surgical or non-surgical treatment of these patients. Contextual factors that influenced the impact of the active disinvestment were lack of communication between stakeholders, others being afraid to lose revenue, patient preferences and other simultaneous interventions.

 A strength of this study is that we investigated how this active disinvestment exercises its effects in daily practice from both organizational and clinical perspectives. As we interviewed all study participants within two years after the active disinvestment was put into place, the results of the present study represent the topical opinions and experiences of key players involved in this process. Our findings add to existing theories on the effect of active disinvestment, which was suggested as a promising alternative to reduce low-value care, regarding the various factors through which the impact in daily practice may be considerably reduced. Limitations of our study include the fact that we did not interview SAPS patients themselves, even though they are important stakeholders in clinical decision-making who are likely to be affected by the active disinvestment initiative.^12^ In that context, it is relevant to note that the preferences of patients were identified as a contextual factor influencing the clinical decision process and indirectly also as factor influencing the effect of the active disinvestment because patients bypass the systems to get their preferred SAD surgery (see Quote 36). Secondly, since we only investigated the active disinvestment initiative for one specific procedure in a Dutch healthcare setting, the results are not necessarily generalizable to other contexts as other factors may be relevant in different circumstances because such initiatives are deemed context-specific.^14^ On the other hand, the overarching themes may still apply, ie, that it should have financial consequences for a hospital to make it worth their effort and that evidence on which it is based should be correctly interpreted, for which it is essential to engage clinicians with relevant expertise. Thirdly, we recruited participants from our professional networks to ensure a diverse sample. It is possible that our professional network does not adequately reflect the views of all sales managers and orthopedic surgeons from the Netherlands. For example, stakeholders who strongly disagree with such active disinvestment initiatives may have been more willing to participate. Furthermore, no orthopedic surgeons from ITCs agreed to participate, while it has been suggested that non-teaching hospitals deliver more low-value care.^33^ We did include participants from non-teaching hospitals as well as a sales manager from an ITC, who will likely capture the main views from ITCs although there may be some context-dependent differences. Additionally, our results may have been biased as the active disinvestment initiative started during the COVID-19 pandemic, so that the active disinvestment may have received less attention from relevant stakeholders within the hospital. Since we provided a clear explanation of the active disinvestment initiative to participants not familiar with the active disinvestment, we do not believe that this had a significant influence.

 Many studies have been published on priority setting and resource allocation in healthcare and de-implementation strategies for low-value care procedures.^34,35^ Hardly any studies have, however, previously evaluated the outcome of an active disinvestment initiative on low-value care.^11,12^ Despite its potentially powerful effect, only few initiatives have shown to result in actual disinvestment.^12^ More frequently, active disinvestment initiatives are preliminary terminated. Rotteveel et al evaluated commonalities between factors influencing the outcomes of active disinvestment initiatives in five recent cases in the Netherlands.^12^ Consistent with the results of the present study, they found that the degree of support from relevant stakeholders largely determined the success of an active disinvestment initiative. Rotteveel et al mainly evaluated the active disinvestment initiatives from a macro-level policy-makers perspective (eg, governmental institutions, health insurers) and concluded that policy-makers should search for interventions for which there is support from relevant stakeholders when applying an active disinvestment initiative. The present study therefore adds evaluating an active disinvestment initiative from a more meso/micro-level perspective (highlighting eg, local institutional factors^36^) and identified several factors at meso/micro-level that contributed to the limited support for active disinvestment from relevant stakeholders. This adds to our understanding how support for active disinvestment initiatives by relevant stakeholders is needed to affect clinical practice, rather than only issuing an active disinvestment by policy-makers without any additional strategies targeting behavioural factors.

 Healthcare providers frequently disagree on how disinvestment initiatives should be prioritized as more than one low-value care practice is often considered suitable for disinvestment. Previous studies on priority setting and de-implementation strategies in healthcare state that the prioritization of such initiatives should also be based on the potential financial impact (ie, cost-saving potential), which is consistent with basic economic theory principles.^37,38^ Our findings are in line with this, as we found that there was no support for the active disinvestment initiative because it had too little financial consequences for the hospital budget given the small number of SAPS patients for most hospitals. Hence, they highlighted the importance of looking for bigger hits with more significant saving potential proportional to the required effort from hospital staff. The importance of a proportional ratio between financial impact and required effort (eg, from hospital staff) is also described by Conrad et al. who explored the effect of monetary incentives on healthcare quality improvement.^39^ They concluded that larger financial incentives are more likely to result in improved quality of care and cover the costs of additional efforts and care process changes.^39^ They also suggested that financial penalties may elicit an even stronger response due to “loss aversion.” However, lack of financial impact may contra wisely limit its effectiveness as it will not motivate healthcare providers to change behaviour nor cover the costs of additional efforts. Furthermore, sales managers related the lack of financial impact of the active disinvestment to it only being a partial reimbursement stop and the active disinvestment being unclear (eg, the use of relative outcome measures) and not specifically enforced. Consequently, orthopedic surgeons could still perform surgery for SAPS and receive reimbursement. It is possible that active disinvestment initiatives would only work in situations with a complete reimbursement stop, as was the case with the successful Vitamin D reimbursement stop in Canada.^11^ Such a complete reimbursement stop makes it impossible to circumvent the active disinvestment. Additionally, it may be that an absolute performance target would have worked better than the relative performance measure used in the active disinvestment regarding SAPS as it is known from literature that these have better incentive properties than relative performance targets.^39^ A difficulty of relative performance targets is that they need adequate baseline measurements, which make clear what proportion of care is of low-value. However, in the active disinvestment regarding SAPS hospitals only knew how many surgical procedures they performed but not whether those were low-value care. Consequently, they did not have a clear view of their improvement potential.

 Orthopedic surgeons felt the healthcare insurer had misinterpreted existing scientific evidence and guidelines on treatment in SAPS patients even though literature suggests that disinvestment initiatives should be based on the strength of evidence supporting the lack of effectiveness.^37^ The construction of evidence in a disinvestment context is a very complex process as the results of scientific evidence are often not “black or white” and subject to between-subject variation in interpretation. Hodgetts et al stated that selection and interpretation of evidence in a disinvestment decision is necessarily framed such that it better fits the disinvestment initiative.^40^ Therefore, they highlighted the need for physician engagement within this process as they can add vital nuance to the debate on what evidence counts in a disinvestment decision and avoid any misinterpretations arising from this ‘fitting it in the disinvestment initiative.’ Additionally, policy-makers often present their disinvestment initiatives as being “black or white” which leaves little room for clinical judgment (it is either low-value care or not) even though it is more nuanced in clinical practice.^41^ Although sometimes there may be clear-cut candidates for disinvestment initiatives, ie, interventions that are entirely ineffective, these are generally scarce as most interventions will have at least some effect or in some situations, as otherwise they would likely have been abandoned already.^41^ Orthopedic surgeons believed that the active disinvestment initiative did not adequately distinguish in the heterogeneous group of etiologies that make up SAPS patients and felt that some patients could still benefit from surgery, which implies that they do not see SAPS as a good disinvestment candidate (ie, may not entirely be low-value care). Although the concept of low-value care is well-known, a clear definition of what constitutes low-value care is missing as well as who decides what constitutes low-value care, which may depend on the perspective taken.^42^ Hence, different stakeholders may have different views on what constitutes low-value care for their situation, as also found in our interviews. In this case, the healthcare insurer decided that most surgery for SAPS patients is low-value care and believed that costs related to this procedure could be saved or should be allocated to other procedures providing more value. The orthopedic surgeons, however, stated that it was not as clear-cut as some patients may benefit from surgery. The latter highlights the tension in perspectives between physicians that want to do as much as possible for their patients and healthcare policy-makers that need to make trade-offs in priority setting in the context of scarcity in healthcare spending.

 There are important implications for future active disinvestment initiatives based on the results from this study, increasing our understanding how active disinvestment initiatives may or may not exercise their effect. The first is that an active disinvestment initiative initiated from a macro-level perspective needs to go together with additional strategies for implementation at micro-level. A crucial step for this implementation at micro-level is to create support from relevant stakeholders,^43^ with the present study identifying several specific factors that may inhibit stakeholders’ support. Although the present study investigated only the perspectives of hospital sales managers and orthopedic surgeons, support from other relevant stakeholders (eg, patients, general public) also have been shown to be essential for successful active disinvestment.^12,44-46^ Gaining support of all relevant stakeholders is, however, extremely difficult as there are often contrasting viewpoints so it will be very complex to design an active disinvestment initiative incorporating all of these views.^41^ In our study, most participants were not against active disinvestment. Still, they highlighted several reasons why they should have been involved from the start in a policy change to (partially) stop reimbursement eg, to ensure correct interpretation of scientific evidence and prioritize initiatives with the most significant cost-saving potential. Such early engagement of relevant stakeholders and transparency of the designing process will therefore create a more nuanced strategy that will enhance the degree of support, thus increasing the possibility of successful active disinvestment.^41,47^ The necessity of stakeholder engagement also has been emphasized in various other studies on eg, priority setting and other de-implementation strategies.^8,43^

 Another implication is that contextual factors will affect the impact of any active disinvestment initiative, such as fear of losing revenues and patient preferences as found in the present study. As a policy change to stop reimbursement will not influence such factors, active disinvestment initiatives should always be paired with other initiatives appealing to the more intrinsic motivation of clinicians such as clinical decision support, performance feedback, patient-oriented educational materials and other interventions that aim to change clinician behaviour.^48-50^ Therefore, future “top-down” policy changes, such as an active disinvestment initiative, should always be combined with “bottom-up” (eg, physician-oriented) co-interventions in order to maximize its effectiveness and increase to possibility for success.^51,52^ Additionally, future active disinvestment initiatives must be aligned with pre-existing theories, such as basic economic theory, and consider theoretical frameworks on eg, priority setting and/or de-implementation^35,53^ as their implications largely overlap. Future research should further explore the effectiveness of active disinvestment initiatives, while taking into account these co-interventions, incorporate the perspectives of patients and develop more specific theoretical frameworks to facilitate understanding how active disinvestment influences (clinical) decision-making. Additionally, future studies must focus on creating a shared view on low-value care and the process around active disinvestment, so that all stakeholders have a uniform perspective in approaching this concept and can start working on initiatives to reduce low-value care.

## Conclusion

 In conclusion, this study showed that two overarching themes negatively influenced the support for and effect of the active disinvestment regarding SAD surgery for SAPS. Hospital sales managers in particular felt it represented a “Too small piece of the pie” while orthopedic surgeons believed “They got it wrong.” Future active disinvestment initiatives should engage all relevant stakeholders at an early stage to gain support, ensure correct interpretation of the evidence and clear definition of the targeted procedures and should target low-value procedures that have sufficient saving-potential to increase the possibility of success.

## Acknowledgements

 We would like to thank all experts who volunteered their time and participated in the interviews.

## Ethical issues

 The study protocol (N20.127) was presented to the Medical Ethical Committee of the Leiden University Medical Center (METC-LDD, Code 058, Leiden, the Netherlands), who waived the need for ethical approval under Dutch law.

## Competing interests

 Authors declare that they have no competing interests.

## Funding

 This work was supported by a grant from ZonMW, the Dutch Organization for Health Research and Development [grant number: 80-83920-98-803]. ZonMW did not influence the study in any way nor the writing of the manuscript.

## Supplementary files

Supplementary file 1. Semi-structured Interview Guides.
