# Supplementary material for: Impact of Active Disinvestment on Decision-Making for Surgery in Patients With Subacromial Pain Syndrome: A Qualitative Semi-structured Interview Study Among Hospital Sales Managers and Orthopedic Surgeons
Source: Int J Health Policy Manag. 2023 Aug 13;12:7710. doi: 10.34172/ijhpm.2023.7710 (PMC10590240; doi:10.34172/ijhpm.2023.7710)
Supplement: Supplementary file 1 — Semi-structured Interview Guides. [file ijhpm-12-7710-s001.pdf]

Article title: Impact of Active Disinvestment on Decision-Making for Surgery in Patients With Subacromial Pain Syndrome: A Qualitative Semi-structured Interview Study Among Hospital Sales Managers and Orthopedic Surgeons

Journal name: International Journal of Health Policy and Management (IJHPM)

Authors' information: Timon H. Geurkink[1,2]*, Perla J. Marang-van de Mheen[2], Jochem Nagels[1], Rudolf W. Poolman[1], Rob G.H.H. Nelissen[1], Leti van Bodegom-Vos[2]

[1]Department of Orthopaedics, Leiden University Medical Center, Leiden, The Netherlands. [2]Department of Biomedical Data Sciences, Medical Decision Making, Leiden University Medical Center, Leiden, The Netherlands.

***Correspondence to:** Timon H. Geurkink, Email: T.H.Geurkink@lumc.nl

Citation: Geurkink TH, Marang-van de Mheen PJ, Nagels J, Poolman RW, Nelissen RGHH, van Bodegom-Vos L. Impact of active disinvestment on decision-making for surgery in patients with subacromial pain syndrome: a qualitative semi-structured interview study among hospital sales managers and orthopedic surgeons. Int J Health Policy Manag. 2023;12:7710. doi:10.34172/ijhpm.2023.7710

Supplementary file 1. Semi-structured Interview Guides

1. Hospital sales managers.

Welcome

Welcome participant and thank participant for participation in interview.

- Verbal consent for participation in study.
- Short personal introduction.
- Characteristics participant

Introduction

- Research about de-implementation of low-value care.
- Research question: effectiveness of an active disinvestment strategy for subacromial pain syndrome.
- Goal interview: to explore how this de-implementation strategy influenced decision-making around surgery from perspectives of relevant stakeholders.

Major topics

- Explanation disinvestment strategy
- Negotiation process between insurers and healthcare providers.
- Attention given to active disinvestment strategy during negotiations.
- Consequences of active disinvestment strategy for hospital.
- Perceived effect of active disinvestment strategy on clinical decision-making

Closing

- Other relevant things to discuss?
- Additional thoughts and/or questions?
- Other relevant stakeholders?

Thank respondent and finish interview

2. Orthopedic surgeons.

Welcome

Welcome participant and thank participant for participation in interview.

- Verbal consent for participation in study.
- Short personal introduction.
- Characteristics participant

Introduction

- Research about de-implementation of low-value care.
- Research question: effectiveness of an active disinvestment strategy for subacromial pain syndrome.
- Goal interview: to explore how this de-implementation strategy influenced decision-making around surgery from perspectives of relevant stakeholders.

Major topics

- Explanation disinvestment strategy
- Treatment strategy for SAPS.
- Perspectives about active disinvestment strategy.
- Perceived effect of active disinvestment strategy on clinical decision-making.
- Other relevant factors.

Closing

- Other relevant things to discuss?
- Additional thoughts and/or questions?
- Other relevant stakeholders?

Thank respondent and finish interview
